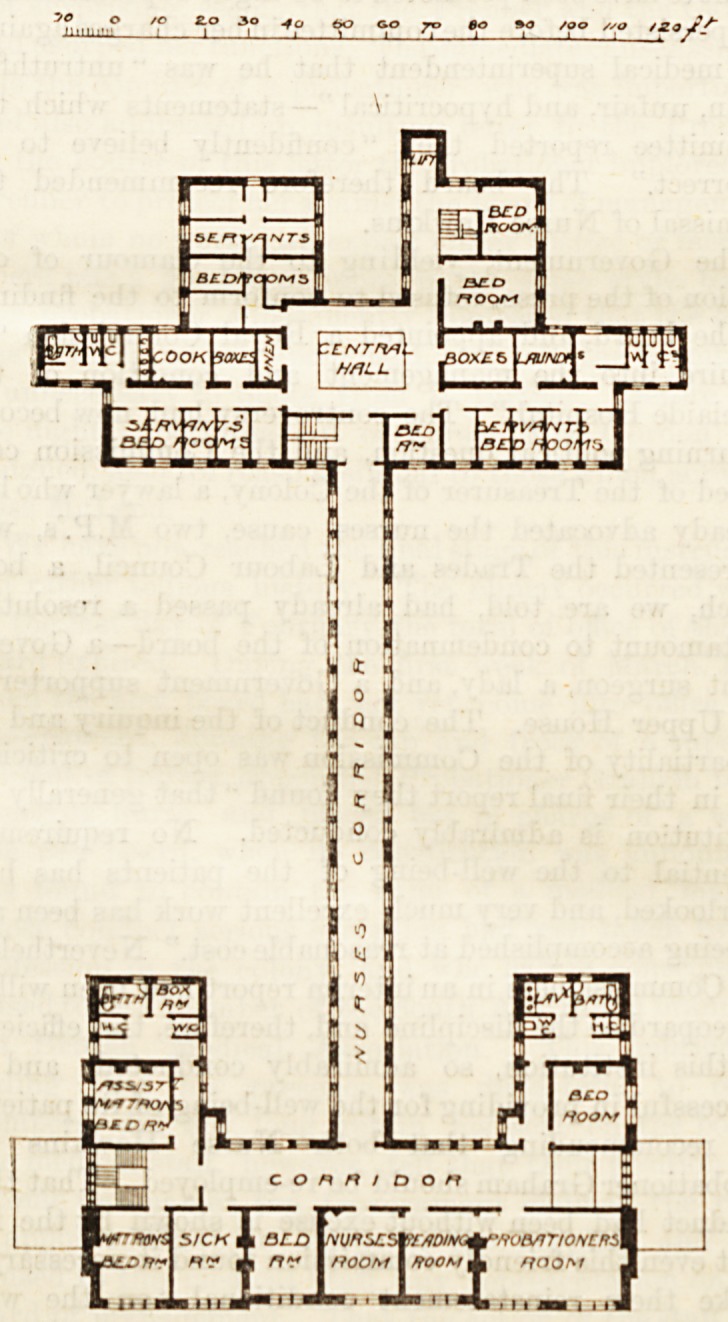# Hospital Construction

**Published:** 1896-10-03

**Authors:** 


### HOSPITAL CONSTRUCTION.

### THE ROYAL HALIFAX INFIRMARY.

The infirmary is placed upon a site of thirteen acres in extent. The whole of it will not be utilised at present, but the plans are so arranged as to permit of future extensions being made as required. The position is open and healthy. The design of the buildings is of a free Renaissance character. The outer walls are "cavity" walls, and have damp courses throughout, and the whole of the buildings, except two floors of the front block, are fireproof. The main frontage has the front administration block in the centre, which is three storeys in height, except at the wings, which are four storied. This block is flanked by detached ward pavilions on both sides.

The chief feature in the general arrangement of the various buildings is that the ward pavilions are only one storey high, and that they are, together with all administrative offices, dining-rooms, kitchens, &c., upon one floor. The ward floors are raised from the ground by means of arches.

The various departments are connected with each other by corridors. The front, or administration, block contains upon the ground floor waiting and secretary's rooms, matron's office and sitting-room, house surgeon's rooms, and board-room, with separate lavatories, &c., in the wings. The complete scheme provides for a nurses' home on the east side, but, as the building of this home is deferred, the nurses are for the present accommodated in the upper floors of the front administration block, in rooms which are both pleasant and commodious, and effectually separated from the hospital proper. The nurses' approach to the wards is by a first floor corridor, from which a staircase descends to the central hall.

The ultimate external effect of the front administration block will be greatly enhanced when the front terrace wall is completed. The design provides for a broad terrace with retaining wall, balustrade, and flight of steps leading to the grounds at the lower end.

The surgical block is a one-storied building at the entrance, to which is a large glass-covered area to shelter carriages, ambulances, &c. This block contains porter's rooms, accident receiving room, examination-rooms, dispensary, &c., and a large hall, which for the present will be used for out-patients. It is proposed, when the whole scheme of the hospital is completed, that an out-patients' department shall be built on the north-west portion of the grounds, at the point nearest to the town.

The central hall, which is at the junction of the two main corridors, is two storeys in height, with a gallery on the first floor. Round it are grouped a number of rooms for the general service of the hospital, comprising assistant matron's office, linen store, sewing-room, and library; also the dining-room for the matron and house-surgeons, and spacious dining-halls for the nurses and servants respectively, all these being provided with pantries. Adjoining the north side of the central hall is an airy and well-lighted service-room communicating with a large and lofty kitchen, adjoining which are a scullery, a pantry, and two store-rooms. Close to the central hall are stairs and a large lift, communicating with the first floor (on which are placed the servants' bed-rooms, box-rooms, lavatories, &c.), and with the basement, which, owing to the levels, forms a ground storey on the lower side. Here are receiving rooms for stores, with goods entrance. The coals are unloaded direct into the coal store from the outside, and taken up to the centre of the building for distribution by a lift provided for this special purpose.

The wash-house and laundry are situated at the rear

PLAN of GROUND FLOOR

WASH HOUSE

LAUNDRY

BOILER HOUSE

LAUNDRY COURT

DYNAMOS

KITCHEN

NURSES MEAL ROOM

SERVANTS HALL

CENTRAL HALL

OPERATION THEATRE

MAIN CORRIDOR

MENS WARD

WOMENS WARD

FUTURE WARD

FUTURE NURSES HOME

DEAD HOUSE

AMBULANCE SHELTER

CORRIDOR

BOARD ROOM

ENTRANCE

of the site, and are separated from the kitchen block by a large grass-covered drying-ground, which will serve as a supplement to the drying arrangements in the laundry. The drying chamber itself is placed between the wash-house and the laundry proper, and is roomy and well lighted.

The administrative parts of the infirmary, as above described, are intended to serve for double the number of patients which are being provided for at present.

The present ward pavilions are six in number, viz., three for men, two for women, and one for children, and will contain 150 beds. Each pavilion is detached and raised from the surface of the ground, and has a cross ventilating passage from side to side at each end of the ward. On one side of the entrance-passage connecting each of the ward pavilions with the main corridor is a small ward for special cases, a patients' clothes-room, and housemaids' closet. On the other side is a nurses' duty-room with pantry, and store-rooms for ward linen, splints, &c. The large wards are all alike, and (except the children's ward) contain twenty beds each. They are 88 ft. long, 28 ft. wide, and 17 ft. high to the centre of the coved ceiling. Four of them have oak floors, the floors of the other two being laid with "Terrazzo" mosaic. All these floors are based on concrete, carried by iron girders. The walls are of Keen's cement, painted with four coats of Gay's enamel paint. All recesses are avoided, and all angles rounded. Each of the large wards is heated by two double fireplaces, and, when needed, by steam heaters, which serve also as inlets for fresh air. At the further end of each ward are lavatories, bath-room, nurses' scullery, &c., and in each of the four wards which face south is a cheerful day-room having an oriel window.

The operating theatre is entered from the main corridor, and in the approach to it are ante-rooms for patients about to undergo operations and for their reception afterwards. The theatre has a good north light, and a floor of white "Terrazzo." The tables are of white glass.

The mortuary is a simple building, well arranged for its purpose, the interior being lined with cream-coloured glazed bricks. Attached are a post-mortem room and a pathological room. The whole of the buildings and grounds are lighted by electricity.

The buildings have been erected according to the designs of Messrs. Worthington and Ellgood, of Manchester, and their successors, Messrs. Thomas Worthington and Son, and under their superintendence.

The cost of the entire works (exclusive of site, and of the boundary wall and lodge, which are left over for the present) is £72,625.

---

## Figures and Tables

**Figure f1:**
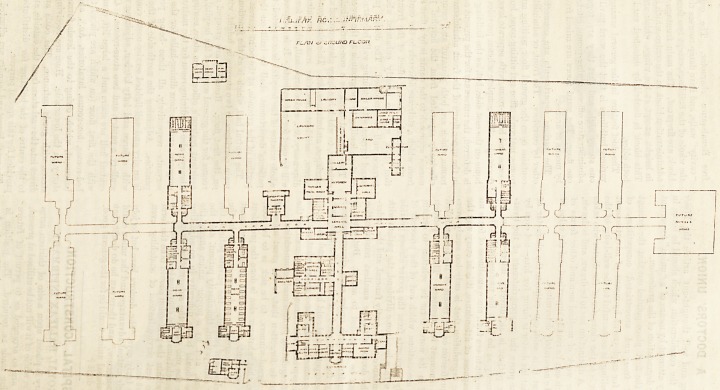


**Figure f2:**